# A forced marriage of IL-2 and PD-1 antibody nurtures tumor-infiltrating T cells

**DOI:** 10.1172/JCI156628

**Published:** 2022-02-01

**Authors:** Erin A. Holcomb, Weiping Zou

**Affiliations:** 1 Graduate Program in Immunology,; 2 Department of Surgery, and; 3 Department of Pathology, University of Michigan School of Medicine, Ann Arbor, Michigan, USA.; 4 Center of Excellence for Cancer Immunology and Immunotherapy, University of Michigan Rogel Cancer Center, Ann Arbor, Michigan, USA.

## Abstract

IL-2 is a pleiotropic cytokine. In this issue of the *JCI*, Ren et al. report on the development of a low-affinity IL-2 paired with anti–PD-1 (PD-1–laIL-2) that reactivates intratumoral CD8^+^ T cells, but not CD4^+^ Treg cells. PD-1–laIL-2 treatment synergized with anti–PD-L1 therapy to overcome tumor resistance to immune checkpoint blockade (ICB) in tumor-bearing mice. Rejection of rechallenged tumors following PD-1–laIL-2 therapy demonstrated the establishment of a potent T cell memory response. Furthermore, PD-1–laIL-2 therapy manifested no obvious toxicity. These findings suggest the potential of PD-1–laIL-2 therapy in treating patients with cancer.

## Targeting IL-2 signaling pathway for cancer therapy

IL-2 is produced primarily by activated CD4^+^ T cells and acts in a paracrine or autocrine fashion (1, 2). IL-2 receptor (IL-2R) signaling occurs through three subunits: alpha (CD25), beta (CD122), and gamma (CD132) (3). Intermediate-affinity dimeric IL-2 receptor consists of IL-2Rβ and IL-2Rγ on naive CD4^+^ and CD8^+^ T cells, memory T cells, and natural killer (NK) cells. TCR engagement or IL-2 stimulation induces the expression of IL-2Rα to form high-affinity trimeric IL-2 receptors that are highly expressed on Treg cells and recently activated effector T cells (4). IL-2 signaling has been an attractive immunotherapeutic target since IL-2 mediates effector T cell activation, including effector CD8^+^ T cells, which are vital for antitumor immunity. High-dose IL-2 was approved by the FDA in 1992 for treatment of certain types of cancer (5). However, IL-2 possesses a very short half-life and requires high doses to be effective, leading to toxicity and severe side effects, such as inflammation and vascular leak syndrome (6). Alternatively, low doses of IL-2 preferentially target IL-2Rα on Treg cells, restricting the immune response, and are associated with poor prognosis in patients with cancer (7, 8). Therefore, methods to target certain T cell subsets while reducing Treg cell binding have been a recent focus in the field of IL-2 therapy.

## Manipulation of T cell phenotype by IL-2 therapy

**Figure 1:**
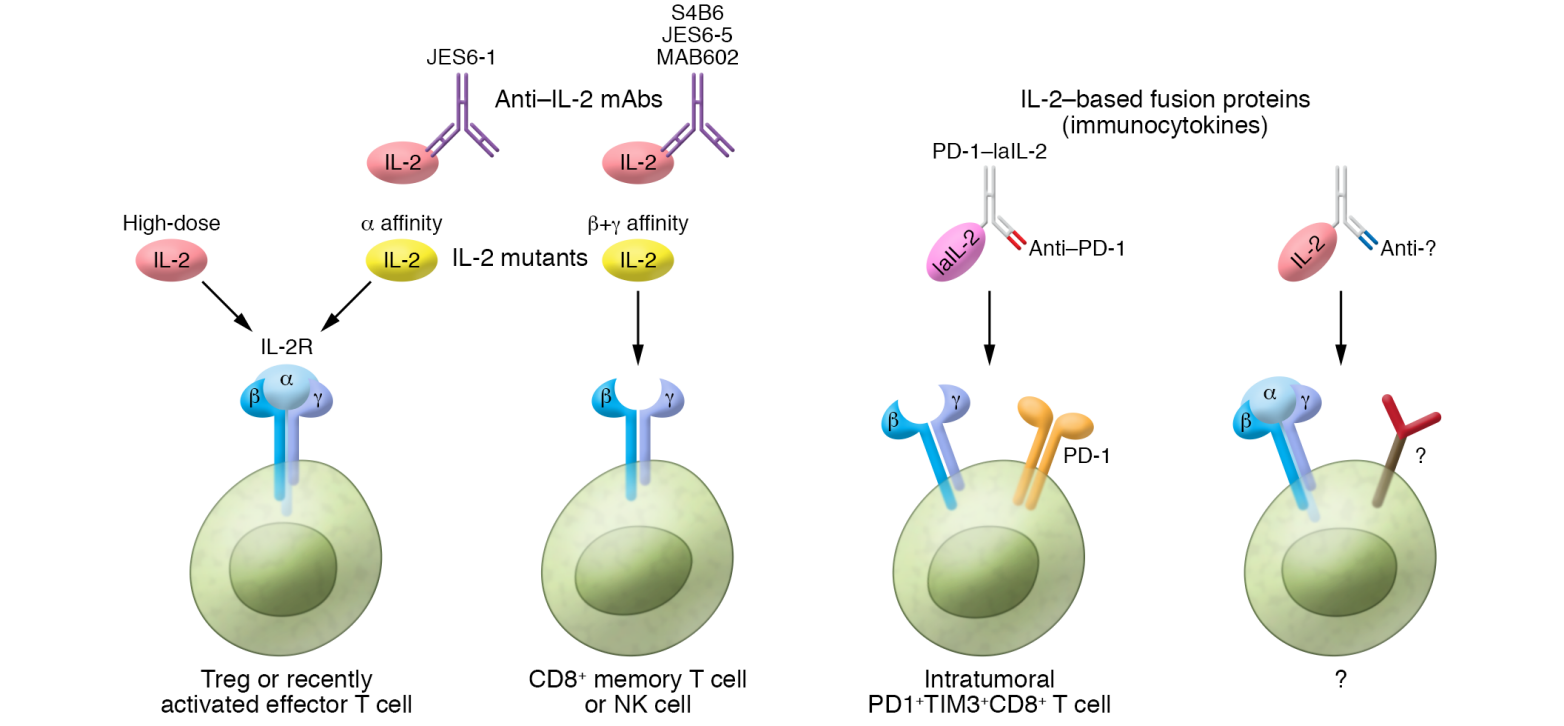
Targeting IL-2 signaling for cancer therapy. High-dose IL-2 may preferentially target high-affinity IL-2R present on Treg cells and recently activated effector T cells. Recent strategies to target IL-2 signaling for cancer therapy include mutant IL-2 with affinity toward different IL-2R chains (alpha, or beta and gamma). Mutant IL-2 with affinity toward IL-2Rα is used to target Treg cells or recently activated effector T cells. Meanwhile, mutant IL-2 with affinity toward IL-2Rβ or IL-2Rγ subunits, rather than IL-2Rα, has been shown to target CD8^+^ memory T cells and NK cells with reduced binding to Treg cells. Combination of IL-2 therapy with various anti–IL-2 mAbs also differentially stimulates specific immune cell subsets. IL-2–based fusion proteins bound to antigen-specific antibodies (immunocytokines) allow for targeted delivery of IL-2 to cells/tissues expressing a protein of interest. PD-1–laIL-2, developed by Ren et al. (20), consists of low-affinity IL-2 (laIL-2) linked to an anti–PD-1 antibody. PD-1–laIL-2 selectively reactivates intratumoral PD-1^+^TIM-3^+^CD8^+^ T cells to enhance antitumor activity. In the future, additional IL-2–based fusion proteins may be engineered to target certain cells of interest in various disease contexts.

To effectively manipulate effector T cells and reduce side effects of high-dose IL-2, IL-2 variants have been developed to stimulate specific T cell subsets through selective targeting of certain IL-2R chains. One strategy has been to introduce mutations in IL-2 to create mutants with preferential IL-2R chain binding. Mutants with reduced IL-2Rβ binding have been shown to target high-affinity IL-2 receptor expressed on effector T cells (Figure 1). These mutants have also exhibited reduced toxicity, possibly due to decreased binding of intermediate-affinity receptors on NK cells that lack IL-2Rα (1, 9). STK-012, a partial IL-2 agonist produced by Synthekine, employs a similar strategy by selectively binding IL-2Rα and IL-2Rβ subunits, but not IL-2Rγ. Effector T cells that may be specific for tumor epitopes can thus expand and readily attack the tumor while avoiding NK cell stimulation (10). However, undesirable Treg cell expansion remains a concern due to high IL-2Rα expression on Treg cells (7, 8). To address this issue, IL-2 mutants with reduced binding to IL-2Rα have also been generated. The cytokine company Nektar has engineered an IL-2 mutant with a bias toward IL-2Rβ and IL-2Rγ, rather than IL-2Rα, to reduce Treg cell binding (10). H9, an IL-2 superkine (sumIL-2) with enhanced IL-2Rβ binding without the need for IL-2Rα, was shown to increase expansion of cytotoxic memory T cells and NK cells while decreasing that of Treg cells (11). Interestingly, H9T, an engineered H9-based partial agonist with further reduced binding to IL-2Rγ, was also recently shown to promote CD8^+^ T cell proliferation that maintained a stem-like memory state and mediated greater antitumor immunity (12).

To enhance the activity of IL-2 in vivo and limit toxicity by reducing the necessary dose, IL-2 therapy has been combined with anti–IL-2 monoclonal antibodies (mAb). Interestingly, various anti–IL-2 mAbs differentially stimulate different immune cell subsets. Anti–mouse IL-2 mAbs S4B6 and JES6-5, as well as anti–human IL-2 mAb MAB602, complexed with recombinant IL-2, selectively stimulate memory CD8^+^ cells and NK cells in vivo to improve IL-2 cancer therapy (Figure 1) (13). On the other hand, anti–IL-2 mAb JES6-1 inhibits proliferation of CD8^+^ cells and NK cells yet maintains its ability to activate Treg cells and has been implicated as a potential treatment for autoimmune disease (14). Binding of these various mAbs to certain regions of IL-2, therefore blocking IL-2 binding to specific IL-2R chains, may explain these contrasting cell type affinities (1, 2).

IL-2–based fusion proteins are another IL-2 therapy strategy with a multitude of current preclinical and clinical trials (15, 16). Fusion of IL-2 to a fragment crystallizable (Fc) region has proven to be beneficial due to increased half-life, complement activation, and induction of antibody-dependent cellular cytotoxicity (ADCC) toward Treg cells (17–19). Furthermore, fusion of IL-2 to antigen-specific antibodies (termed an immunocytokine) allows for targeted delivery of IL-2 to cells and tissues expressing a protein of interest. Numerous IL-2 immunocytokines have been developed to target tumor-associated antigens expressed by cancer cells and their surrounding tissue (16). IL-2 is therefore honed to tumor tissues to enact its function. However, this strategy still lacks the ability to specifically target effector T cells within the tumor that are pertinent to anticancer immunity.

## Targeting intratumoral effector T cells with IL-2 and anti–PD-1 therapy

Ren et al. (20) addressed this intratumoral T cell targeting gap by engineering an immunocytokine fusion protein consisting of low-affinity IL-2 (laIL-2) linked to an anti–PD-1 antibody (PD-1–laIL-2). laIL-2 exhibits reduced binding to IL-2Rα and IL-2Rβ to diminish unfavorable Treg cell binding in the tumor and periphery. Meanwhile, PD-1 is highly expressed on tumor-infiltrating CD8^+^ T cells. As a result, PD-1–laIL-2 possessed elevated avidity toward intratumoral CD8^+^ T cells, rather than Treg cells or peripheral CD4^+^ and CD8^+^ T cells. This specificity not only reduced the systemic toxicity, but also enhanced tumor control in A20 and MC38 tumor models, as well as A375 tumor-bearing humanized mice. In addition, PD-1–laIL-2 in combination with anti–PD-L1 therapy overcame tumor resistance to PD-L1 blockade therapy. Notably, this effect was dependent on intratumoral CD8^+^ T cells, whose proliferation was selectively induced by PD-1–laIL-2. Further investigation revealed that PD-1–laIL-2 seemed to selectively target intratumoral PD-1^+^TIM-3^+^CD8^+^ T cells, which are usually described as a functionally exhausted and/or terminally differentiated T cell subset. Therefore, PD-1–laIL-2 could reactivate PD-1^+^TIM-3^+^CD8^+^ T cells to enhance antitumor activity (Figure 1). Tumor rechallenge resulted in spontaneous rejection in tumor-bearing mice previously treated with PD-1–laIL-2. This effect was also dependent on the presence of CD8^+^ T cells, indicating these rejuvenated T cells are tumor antigen-specific and can mediate a strong memory response. These promising results suggest that PD-1–laIL-2 therapy may bring clinical benefits to patients with cancer.

## Version 1 02/01/2022

Electronic publication
